# Left Prefrontal Activity Reflects the Ability of Vicarious Fear Learning: A Functional Near-Infrared Spectroscopy Study

**DOI:** 10.1155/2013/652542

**Published:** 2013-11-05

**Authors:** Qingguo Ma, Yujing Huang, Lei Wang

**Affiliations:** ^1^Neuromanagement Laboratory, Zhejiang University, Hangzhou 310028, China; ^2^School of Management, Zhejiang University, No. 38 Zheda Road, Hangzhou 310028, China

## Abstract

Fear could be acquired indirectly via social observation. However, it remains unclear which cortical substrate activities are involved in vicarious fear transmission. The present study was to examine empathy-related processes during fear learning by-proxy and to examine the activation of prefrontal cortex by using functional near-infrared spectroscopy. We simultaneously measured participants' hemodynamic responses and skin conductance responses when they were exposed to a movie. In this movie, a demonstrator (i.e., another human being) was receiving a classical fear conditioning. A neutral colored square paired with shocks (CS_shock_) and another colored square paired with no shocks (CS_no-shock_) were randomly presented in front of the demonstrator. Results showed that increased concentration of oxygenated hemoglobin in left prefrontal cortex was observed when participants watched a demonstrator seeing CS_shock_ compared with that exposed to CS_no-shock_. In addition, enhanced skin conductance responses showing a demonstrator's aversive experience during learning object-fear association were observed. The present study suggests that left prefrontal cortex, which may reflect speculation of others' mental state, is associated with social fear transmission.

## 1. Introduction

Fear can be learned indirectly by observing others behaving fearfully. When shown a demonstrator's fearful reactions to a neutral stimulus, observers rapidly learn fears to the neutral stimulus later presented alone and show all of Lang's response systems [1], including stronger fear beliefs, increased physiological responses, and overt avoidance behaviors [2–5]. Although the classical fear conditioning is well understood [6–10], little is known about the mechanism of observational fear learning. 

There are several possible routes to fear learning by-proxy. First, vicarious learning is procedurally the same as classical fear conditioning [2, 5]. During this procedure, observers form an association between a neutral stimulus and an aversive event (e.g., an electric shock) to a demonstrator [11] or form an association between a neutral stimulus and a transmitted social cue (e.g., demonstrator's fearful expression) [12]. Second, the observers show “empathy” towards the behaviors of the demonstrator. An increasing number of studies examined the relationship between empathy and vicarious fear [13–15]. 

An additional challenge is to assess the neural mechanism of observational fear learning. Some functional magnetic resonance imaging (fMRI) studies [16, 17] suggest that observational learning involves the same neural pathway as learning from direct experience. Specifically, amygdala-hippocampal complex is active when learning the association between fear and the neutral stimulus via observing someone else's fearful responses [16]. However, other studies [18–20] indicate that observational fear learning is partly different from classical fear learning. It is suggested that the anterior cingulate cortex (ACC) [18, 20] and medial prefrontal cortex (mPFC) [19, 20], which are examined by vast literature to be related to mentalizing others' states [21, 22], are involved in social transmission of fear. 

The aim of the current study was to investigate empathy-related processes in functional prefrontal activation patterns during observational fear learning by using functional near-infrared spectroscopy (fNIRS). fNIRS is a noninvasive neuroimaging technique and can detect the changes of oxygenated and deoxygenated hemoglobin concentration in brain. In addition, fNIRS has the potential for real-time measurement even when there is muscular activity [23]. According to the tight coupling of oxygen delivery and neural activity [24], both decrease in deoxygenated hemoglobin concentration and increase in oxygenated hemoglobin concentration are taken as indicators of cortical activation [25]. We hypothesized that when subjects were watching a demonstrator who was seeing a stimulus paired with traumatic events, the activities of their prefrontal cortex (PFC), indexed by the concentration changes of oxygenated hemoglobin, would have increased compared to that of a stimulus unpaired with aversive events. In addition, skin conductance response (SCR), which represents a relatively late biological response [26, 27], was also measured during the observational fear learning. We further predicted that enhanced SCR during fear learning by-proxy was observed. 

## 2. Materials and Methods

### 2.1. Subjects

Thirty-one healthy right-handed subjects were recruited (15 females, mean age = 22.4 years, aged 19–26 years). All participants reported normal or corrected-to-normal vision and had no history of neurological or psychiatric disorders. All subjects were given informed consent, which was approved by the Institutional Ethical Committee of School of Management at Zhejiang University. All participants were paid for their participation. Research was carried out according to the principles of the Declaration of Helsinki.

### 2.2. Procedures

**Figure 1:**
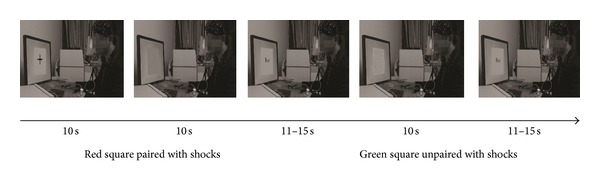
Experimental design. Participants watched a demonstrator responding to colored squares paired with shocks and the other type of colored squares unpaired with shocks.

We made a movie (180 seconds in total) which showed a male demonstrator, with fNIRS and SCR recording sets on the head and the back of right hand, respectively. A device with the capacity to send shocks was put on the back of left hand. The demonstrator was participating in a classical fear conditioning experiment. In front of this demonstrator, one of two colored squares (green or red) was randomly presented. The duration of each colored square was 10 seconds. The squares were intervened by an image of the word “Rest” with an interval of 11–15 seconds. One type of colored squares was paired with uncomfortable shocks to the demonstrator. The shocks would occur randomly 60% of times. The other type was never paired with shocks. Each type of colored squares was presented for five times. Participants sat comfortably and were provided with the same stimulation device as the demonstrator. Subjects were instructed to watch this movie. After observation, participants would receive the same procedure as the demonstrator. The experimental procedure during the movie projection was shown in Figure 1.

### 2.3. SCR Analyses

SCR was picked up from index and middle finger of the subject's right hand by employing the equipment of NeXus-10. Based on the trough-to-peak analysis [28], the amplitudes of SCR were calculated by predefining response window (1–5 s) after stimulus onset. Then SCR was computed as the conductance changes of onset and peak time, which were then converted to logarithmic values by adding 1 and then multiplying by 1000 [29]. Using paired-sample *t*-test, we compared the differences of mean amplitudes of SCR between CS_shock_ and CS_no-shock_ during the movie projection.

### 2.4. fNIRS Measurements and Analyses

**Figure 2:**
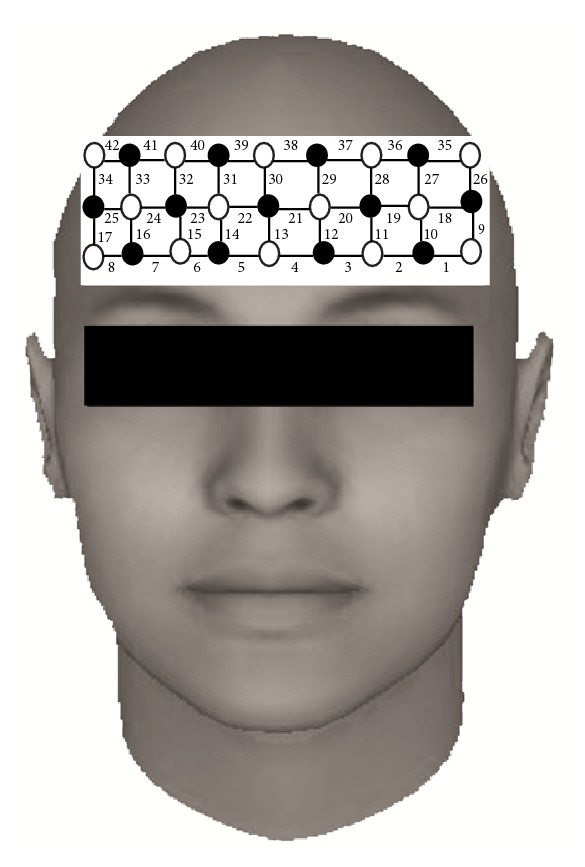
Location of the 42 channels with the 3 × 9 probe holder. Circles are emitters (open) and detectors (closed). The numbers indicate channels' number.

We used a continuous wave-type fNIRS system (FOIRE 3000/16; Shimazu Co., Japan), which emitted three near-infrared lights (wavelengths, 780 nm, 805 nm, and 830 nm) at each source fiber. As shown in Figure 2, a single 3 × 9 optode probe with 42 channels was fixed by a holder cap around the forehead of both hemispheres. The detectors and sources were alternately placed at an equal distance of 3 cm. The sampling rate was approximately 11.76 Hz; hence, the time gap between sample points of each detector for oxygenated hemoglobin (HbO) and deoxygenated hemoglobin (HbR) signals was 85.03 ms. We used a 3D digitizer to localize the measured fNIRS channels in Montreal Neurological Institute (MNI) space and presented these channels on an anatomical image [30].

We used NIRS-SPM, a statistical parametric mapping (SPM) based on MATLAB software [31], to analyze fNIRS data. fNIRS intensity measurements at three wavelengths were converted to relative changes in HbO and HbR using the modified Beer-Lambert law. Beer-Lambert law assumed that there was a linear relationship between the absorption of electromagnetic radiation and the concentration of scattering tissue in a given medium [32]. The wavelet-minimum description length based detrending algorithm was applied to eliminate global drift due to breathing, cardiac motion, body motion, and so on. This detrending algorithm played a role in decomposing fNIRS measurements into global trends, hemodynamic signals, and uncorrelated noise components at distinct scales [33]. Epochs were segmented from the stimuli onset to 10 s later for each trial. We expected that the activation peak for the experimental conditions appeared within this temporal window. Mean concentration changes of HbO and HbR were extracted for each trial, channel, and participant during the movie projection. Paired-sample *t*-test for individual level and group level was used to analyze mean value over trials for each channel. The independent variable was the type of colored squares during watching the movie: CS_shock_ versus CS_no-shock_. 

## 3. Results

### 3.1. SCR Results

**Figure 3:**
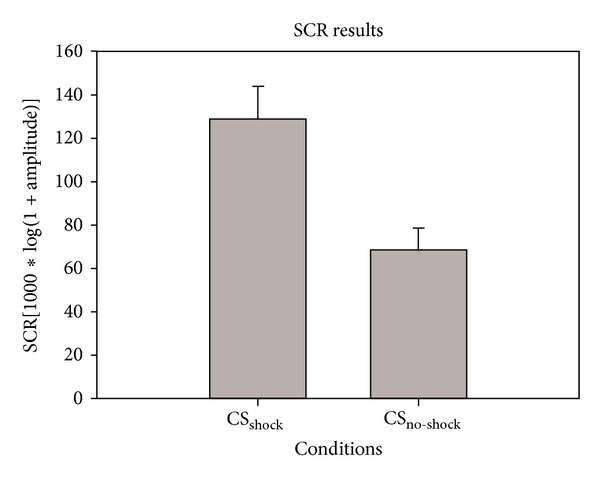
SCR results. *x*-axis indicates independent variables: CS_shock_ versus CS_no-shock_. *y*-axis reflects the mean amplitudes of SCR calculated from all subjects (mean ± S.E.). The average SCR of CS_shock_ was larger than that of CS_no-shock_ during the movie projection.

The results showed that mean amplitudes of SCR were larger (*t*_30_ = 5.71, *P* < 0.001) when participants were watching a demonstrator seeing CS_shock_ (Mean = 128.56, S.D. = 85.44) than that exposed to CS_no-shock_ (Mean = 67.90, S.D. = 59.11) (see Figure 3). 

### 3.2. fNIRS Results

**Figure 4:**
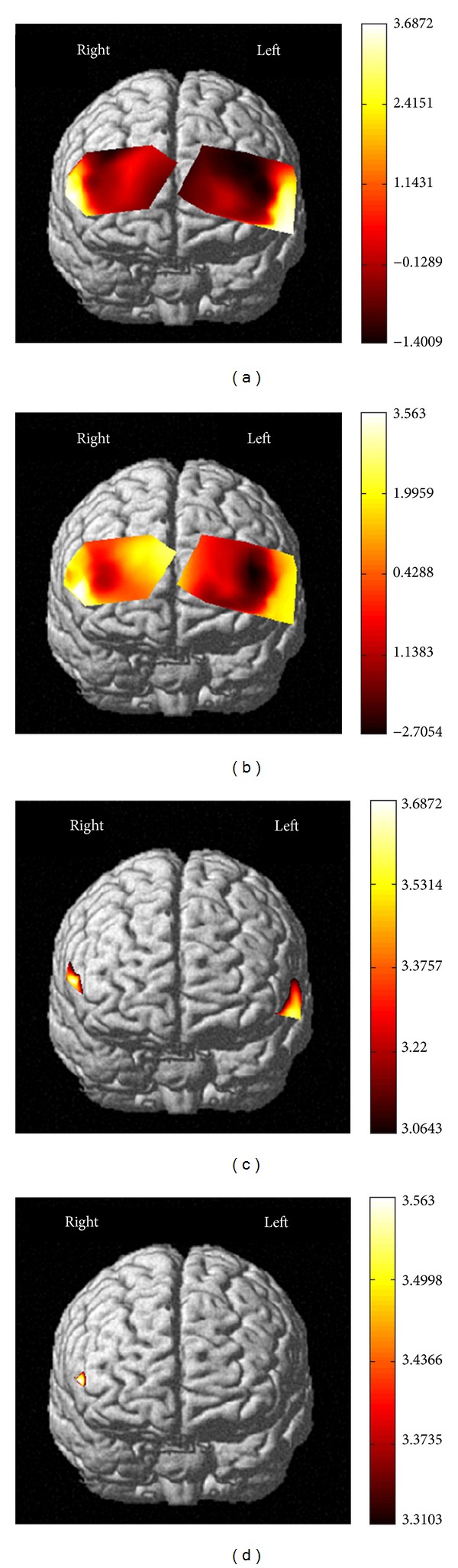
Individual maps. Individual *t*-statistic maps from (a) HbO and (b) HbR. Individual activation maps from (c) HbO and (d) HbR.

For calculating *t*-values, NIRS-SPM software was used. NIRS-SPM allowed the estimation of the temporal correlation, determined a Lipschitz-Killing curvature based (LKC-based) expected Euler characteristics corrected *P* value; and obtained both individual and group *t*-statistic maps using the classical interpolation method. For more detailed discussion on NIRS-SPM, previous work [29, 31, 33] could be referred to. For the individual session, individual's *t*-statistic maps were obtained (Figures 4(a)-4(b)) to compare concentration changes of HbO and HbR between CS_shock_ and CS_no-shock_. Besides, for the same participant, individual activation maps (CS_shock_ minus CS_no-shock_) were acquired for HbO and HbR (Figures 4(c)-4(d)). The degree of freedom was 85.2421, *P* < 0.05. LKC-based expected Euler characteristics correction was used. The top four *t*-value channels of HbO were channel 1 (*x* = −53, *y* = 26, *z* = −8, and *t* = 3.395), channel 9 (*x* = −64, *y* = 0, *z* = −9, and *t* = 3.5145), channel 16 (*x* = 56, *y* = 40, *z* = 8, and *t* = 3.4153), and channel 25 (*x* = 59, *y* = 32, *z* = 9, and *t* = 3.6872). In addition, the top three *t*-value channels of HbR were channel 7 (*x* = 51, *y* = 50, *z* = 0, and *t* = 2.8019), channel 16 (*x* = 56, *y* = 40, *z* = 8, and *t* = 3.5423), and channel 42 (*x* = 60, *y* = 23, *z* = 24, and *t* = 2.9085). 

**Figure 5:**
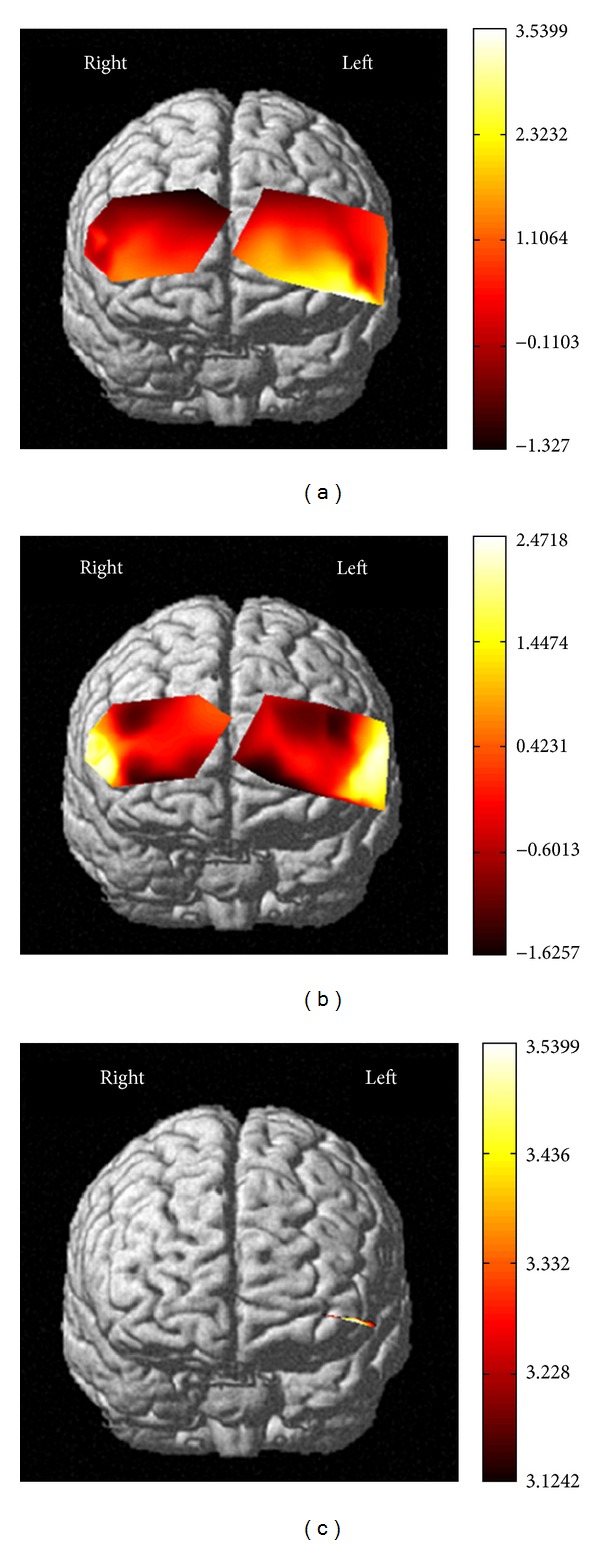
Group maps. *t*-statistic maps from (a) HbO and (b) HbR obtained by group analysis. (c) Activation map from HbO found by group analysis using the LKC-based expected EC method (31 subjects, corrected *P*-value < 0.05).

To compare concentration changes of HbO and HbR between CS_shock_ and CS_no-shock_ across individuals, NIRS-SPM was used to obtain the group *t*-statistic map from HbO and HbR (Figures 5(a)-5(b)).  Figure 5(c) illustrated activation map from HbO during observation. The total number of subjects used in the group analysis was thirty-one. We used LKC-based expected Euler characteristics correction method. Activation region (CS_shock_ minus CS_no-shock_) found by group analysis of HbO was shown in Figure 5(c) (corrected *P* value < 0.05). The activated region found by HbO signal was roughly localized to the dorsolateral prefrontal cortex. We found no activation region by group analysis of HbR (corrected *P*-value < 0.05). 

## 4. Discussion

The present study investigated the neural mechanism of observational fear learning by measuring concentration changes of HbO and HbR. Accordingly, it can be hypothesized that observers were likely to keep the prefrontal cortex recruited during learning fear from a demonstrator. Specifically, increased HbO concentration in left PFC was found when participants were watching a demonstrator seeing a neutral stimulus paired with a shock. In addition, the present study replicated earlier work and found that enhanced SCR was apparent when observers witnessed a demonstrator's traumatic experience. 

Enhanced SCR in our study validated the extension of traditional fear conditioning models to vicarious fear learning [2, 3, 5]. The initial experiments to explore the social fear conditioning in controlled laboratories verified that fear responses, such as self-report beliefs and avoidance behaviors, could be acquired via observation [2, 3, 5, 12]. However, this work was mainly criticized for (1) looking into self-report fear beliefs and failing to acquire physiological fear and (2) saying little about the neural mechanism of fear learning [20, 26]. Thus, SCR was a widely used measure of states of arousal [34] and had been successfully employed in vicarious fear learning. For example, Berger (1962) found participants showing enhanced SCR when a model showed fearful expression during observation [27]. Besides, Olsson and Phelps (2004) also demonstrated a comparable difference of SCR between conditioned stimuli and unconditioned stimuli in a social fear transmission task [26]. In agreement with prior studies, the enhanced SCR in our study might be related to increased arousal of participants when they witnessed a demonstrator watching colored squares paired with traumatic events. 

Increased concentration of HbO in left PFC was observed when participants were shown a demonstrator seeing colored squares paired with shocks. This fNIRS results demonstrated that observational fear learning might rely on some brain areas which were different from classical fear learning. Previous fMRI studies [16, 17, 35] suggested that observational fear learning used the same neural mechanism as classical fear learning. Specifically, the amygdala-hippocampal complex was more active when learning an association between a neutral object and a demonstrator's aversive experience than a neutral object and a demonstrator's safe experience [16]. However, with the source detector separation (3 cm) used in the fNIRS device, the penetration depth of the near-infrared light in participant's head was limited to the surface of the cortex only [36]. Moreover, the device used in the current study was designed to be applied only on the forehead [37]. Although observational fear learning involved amygdala and hippocampus [16], the brain activity in our study was documented only from the prefrontal cortex. 

It was suggested that the region of PFC contributed to mentalizing others' states [38–41] and introspecting about self [42–44]. The increased concentration of HbO may cautiously explain that participants learned fear from the demonstrator by empathizing with others' mental states. Consistently, the relationship between empathy and observational learning has been explored in recent decades. For example, one study [14] showed that when housing the observing triads and demonstrating triads together for a long period of time, the observing rodents' anxiety level was higher during observational fear learning than when the two groups of triads were housed independently. This finding illustrated that empathy between observing rodents and demonstrating rodents may contribute to the effect. Similarly, it has been proved that the observer's capacity of empathizing with the demonstrator might influence the experience distress [15]. Besides, Colloca and Benedetti (2009) found that placebo analgesia responses induced after social observational learning were positively correlated with empathy scores of the empathy questionnaire, suggesting that empathic concern may have modulated social fear transmission [13]. Recently, an fMRI study conducted by Olsson et al. (2007) on vicarious fear learning [20] reported the important role of PFC in mentalizing others' fear responses during learning object-fear association. Our results were in line with previous studies and demonstrated that, during learning fear via observation, empathy-related brain regions played a role in establishing the object-fear association. Observers may need to mentalize others' states during learning fear via observation.

The fNIRS analysis revealed that the concentration changes of HbO were roughly located in the left PFC rather than bilateral PFC. It may be attributed to the participants' age. Prior fMRI researches [45–50] have suggested that, with the life stage (childhood, adolescence, and adulthood), the lateral PFC activation becomes larger. Besides, one fNIRS study [51] found that old adults showed bilateral PFC activity during all N-back working memory task, while young adults showed slight right-hemispheric dominance during 0-back and 1-back performance. Although the function of age-related reductions in PFC activation asymmetry was unclear, it could be interpreted that young people may need to recruit less cortical regions, but old adults need to compensate for reduced neural efficiency [52]. In our study, we recruited volunteers ranging from 19 to 26 years old, who may not need to elicit bilateral PFC in this task.

Our study indicated that observational fear learning involved the concentration changes of HbO, which could be attributed to the changes in neurovascular coupling during social fear learning. Neurovascular coupling referred to the relationship between local neural activity and subsequent changes in hemodynamic properties of the surrounding vasculature, including cerebral blood volume, cerebral blood flow, and cerebral metabolic rate of oxygen [53]. The increased oxygenated hemodynamic concentration changes in PFC for CS_shock_ condition can be interpreted as an increase in oxygen consumption by neurons when participants speculated others' states. fNIRS was sensitive to hemodynamic changes at the capillary level rather than those at the small venous vessel level [35]. Accordingly, fNIRS may be a valuable complementary method to fMRI in unraveling the neural mechanism of fear learning by-proxy.

In our study, we found no significantly different changes of HbR by group analysis. This was in line with some previous fNIRS studies [35, 53], which held the notion that concentration changes of HbO were more sensitive to neural activation compared with changes of HbR [35]. Thus, we affirmed that HbO might be a more robust indicator for changes in regional cerebral blood flow, due to larger changes in amplitude. Hence, the changes of HbO rather than changes of HbR may be used to understand the PFC activation in social fear transmission.

There are several limitations in our study. First, although the observational fear learning highly involved amygdala-hippocampal complex during learning object-fear association, the current study's setup cannot illustrate the relationship between these regions and frontal lobe. Second, the moderate number of participants might attenuate the effect of experimental task. Thus, these analyses might not strictly rule out possible effects of unavoidable variables. 

In conclusion, the activation of PFC, indexed by concentration changes of HbO during observational fear learning task, suggests that social fear learning is partly different from classical fear learning. Our results indicate that empathy-related brain regions may contribute to fear learning processes via observation. This study extends previous research by employing fNIRS and confirms that vicarious learning is a viable pathway through which fear transmits in social context.
